# Monocyte TREM-1 Levels Associate With Anti-TNF Responsiveness in IBD Through Autophagy and Fcγ-Receptor Signaling Pathways

**DOI:** 10.3389/fimmu.2021.627535

**Published:** 2021-03-15

**Authors:** Marileen M. Prins, Bram Verstockt, Marc Ferrante, Séverine Vermeire, Manon E. Wildenberg, Pim J. Koelink

**Affiliations:** ^1^ Tytgat Institute for Liver and Intestinal Research, Amsterdam UMC, Location AMC, Amsterdam, Netherlands; ^2^ Department of Gastroenterology and Hepatology, University Hospitals Leuven, KU Leuven, Leuven, Belgium; ^3^ Translational Research Center for Gastrointestinal Disorders [TARGID], Department of Chronic Diseases, Metabolism and Ageing [CHROMETA], KU Leuven, Leuven, Belgium; ^4^ Department of Gastroenterology and Hepatology, Amsterdam UMC, Location AMC, Amsterdam, Netherlands

**Keywords:** anti-TNF response, IBD, TREM-1, Fc receptor, autophagy

## Abstract

The expression of *Triggering Receptor Expressed on Myeloid cells* (*TREM)-1* has been described as a predictive marker for anti-Tumor Necrosis Factor (TNF)-α monoclonal antibody (mAb) therapy responsiveness in patients with inflammatory bowel disease (IBD). Here we investigated expression of *TREM-1* specifically in CD14+ monocytes in relation to anti-TNF response. The pretreatment *TREM-1* expression levels of CD14+ monocytes of Crohn’s disease (CD) patients were predictive of outcome to anti-TNF mAb therapy, with low *TREM-1* expression associated with response to anti-TNF. FACSorting of CD14+ monocytes with different TREM-1 levels showed that differentiation towards regulatory CD206+ M2 type macrophages by anti-TNF was suppressed in CD14+ monocytes with high TREM-1 expression. Activity of the Fcγ-Receptor and autophagy pathway, both necessary for M2 type differentiation and the response to anti-TNF, were decreased in CD14+ monocytes with high expression of TREM-1. We confirmed that the activity of the Fcγ-Receptor pathway was decreased in the CD patients that did not respond to anti-TNF therapy and that it was negatively correlated with *TREM-1* expression levels in the CD patient cohort. In conclusion, our results indicate that *TREM-1* expression levels in CD14+ monocytes associate with decreased autophagy and FcγR activity resulting in decreased differentiation to M2 type regulatory macrophages upon anti-TNF mAb treatment, which may explain anti-TNF non-response in IBD patients with high expression levels of *TREM-1*.

## Introduction

Despite the considerable efficacy of anti-Tumor Necrosis Factor (TNF)-α monoclonal antibody (mAb) therapy in the treatment of inflammatory bowel disease (IBD), about one third of patients do not respond (1). The reason for this primary non-response is not entirely understood, therefore the search for predictive markers has been hampered considerably (2). In latest years, several research groups described the association of the expression levels of several genes in the intestine in relation to anti-TNF response in IBD (3–8). However due to the invasiveness of intestinal biopsies and patient’s preference prognostic tests on blood samples would be favorable for clinical practice. Therefore, after determining the association of the intestinal expression of *Triggering Receptor Expressed on Myeloid cells* (*TREM)-1* with response to anti-TNF therapy, Gaujoux et al. (3) investigated the association of whole blood *TREM-1* expression and anti-TNF response, and reported that low whole blood *TREM-1* levels predict anti-TNF non-responsiveness. In contrast, Verstockt et al. (9, 10) reported exactly the opposite, high whole blood *TREM-1* levels predict anti-TNF non-responsiveness. Also an association was found between the soluble TREM-1 (sTREM-1) levels in serum and anti-TNF response, with high levels being associated with anti-TNF non-response (9). Although the studies defined response to anti-TNF differently, the explanation for the opposite association with anti-TNF response was not completely clear.

Over the last decade we have shown that anti-TNF mAbs interact with Fc Receptors on myeloid cells, and that this interaction is crucial for the therapeutic efficacy in IBD (11–13). Through this Fc-Receptor interaction anti-TNF mAbs skew monocytes towards CD206+ macrophages (13, 14), exhibiting increased levels of autophagy (15). As TREM-1 is selectively expressed on monocytes and neutrophils in whole blood (16), and monocytes/myeloid cells are critical in the response to anti-TNF (11, 13, 14, 17), we investigated whether the *TREM-1* expression in CD14+ monocytes is related to future anti-TNF response, and what could be the functional link between the two.

## Material and Methods

### Human Cell Isolation and Cultures

Peripheral blood mononuclear cells (PBMCs) from healthy volunteers (with written informed consent (Amsterdam UMC, METC 2009_113) were isolated by Ficoll Paque density-gradient centrifugation. After washing, monocytes were isolated by Percoll density-gradient centrifugation. TREM-1 low and high sorted CD14+ monocytes were cultured in RPMI supplemented with 10% heat-inactivated FCSin a 1:5 ratio with untouched T-cells isolated from PBMCs of a different donor by negative isolation (Dynabeads Untouched Human T Cells #11344D, Invitrogen). After 2 days the full monoclonal anti-TNF adalimumab (Humira^®^, AbbVie, Wavre, Belgium) or isotype control IgG1 (GTX16193, Genetex) was added (both 10 µg/ml) and cultured for another 3 days. Similar, in the mixed lymphocyte reaction (MLR) PBMCs of two different donors were mixed in a 1:1 ratio and the agonistic mouse monoclonal TREM-1 antibody (Clone#193015, R&D systems) or isotype control (Clone#11711, R&D systems), both 10 µg/ml, was added together with anti-TNF or IgG after two days. In the MLR with the TREM-1 inhibitor LR12 (LQEEDAGEYGCM, >98% purity, Pepscan, Lelystad), twelve hours before adding anti-TNF (after 2 days of culture) LR12 (50 µg/ml), was added. Upon the addition of anti-TNF or isotype control (10 µg/ml) the LR12 was refreshed and every 24 hours afterwards until a total of 5 days of culture. 6-thioguanin (6TG, 25 µM) was dissolved in dimethylsulfoxide (DMSO) and added together with the anti-TNF with DMSO as control as reported before (18). Monocytes were cultured for 24 hours with or without LPS (100 ng/ml) in combination with LR12 or the agonistic TREM-1 antibody and TNFα production was measured in supernatant by ELISA (DY210, R&D systems).

### Crohn’s Disease Patient Study

All patients had given written consent to participate in the Institutional Review Board approved IBD Biobank of University Hospitals Leuven, Belgium (B322201213950/S53684), Twenty-four anti-TNF naïve patients with Crohn’s disease (CD), initiating anti-TNF therapy because of active endoscopic disease (presence of ulcerations), were enrolled. Prior to treatment initiation, a 20 ml blood sample was taken, and PBMCs were isolated by density centrifugation. After cell isolation, samples were cryopreserved with DMSO using Mr Frosty (Thermo Fisher Scientific, Waltham, Massachusetts, USA) for 24 hours and afterwards stored in liquid nitrogen. Endoscopic remission was assessed at 6 months after therapy initiation, and defined as a complete absence of ulcerations (19). Patient characteristics (17 responders, 7 non-responders) are depicted in **Table 1**.

**Table 1:** Baseline characteristics of the Crohn’s disease patients.

	Responders (n = 17)	Non-responders (n = 7)
Gender, women, *n* (%)	5 (29.4)	3 (42.9)
Disease duration, *y*, median (IQR)	4.0 (0.9 – 13.6)	4.6 (0.5 – 18.0)
Age at inclusion, *y*, median (IQR)	30.2 (22.9 – 33.0)	44.0 (26.4 – 53.9)
C-reactive protein, *mg/L*, median (IQR)	6.7 (2.5 – 20.5)	7.9 (4.9 – 11.4)
Serum albumin, *g/L*, median (IQR)	40.3 (37.8 – 45.0)	39.3 (38.4 – 43.1)
Disease location, *n* (%)		
- Ileal disease (L1)	3 (17.6)	3 (42.9)
- Colonic disease (L2)	5 (29.4)	2 (28.6)
- Ileocolonic disease (L3)	9 (52.9)	2 (28.6)
- Upper GI involvement (L4)	1 (5.9)	1 (14.3)
Disease behaviour, *n* (%)		
- Inflammatory (B1)	11 (64.7)	2 (28.6)
- Stricturing (B2)	3 (17.6)	3 (42.9)
- Penetrating (B3)	3 (17.6)	2 (28.6)
- Perianal disease (p)	6 (35.3)	1 (14.3)
Concomitant medication, *n* (%)		
- Corticosteroids		
• Topical steroids	4 (23.5)	0 (0.0)
• Systemic steroids	3 (17.6)	1 (14.3)
- Immunomodulators	11 (64.7)	3 (42.9)
Smoking status, *n* (%)		
- Active smoking	3 (17.6)	1 (14.3)
- Previously smoking	3 (17.6)	2 (28.6)
- Never smoked	11 (64.7)	4 (57.1)

### Flow Cytometry

**Figure 1:**
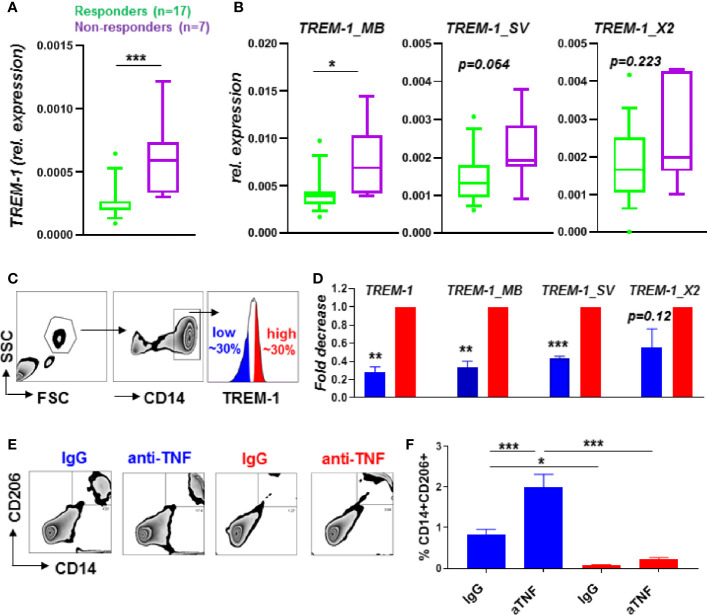
TREM-1 levels in CD14+ monocytes are low in anti-TNF responders associated with a decreased M2 macrophage polarization by anti-TNF. Total *TREM-1* expression **(A)** and the different TREM-1 isoforms *TREM-1_MB, TREM-1_SV* and *TREM-1_X2* **(B)** expression levels in CD14+ monocytes from CD patients (n=24) before the start of anti-TNF therapy, and FACsorted (sort strategy in **(C)**) CD14+ monocytes (n=4) with different TREM-1 levels **(D)**. Expression relative to *bACTIN* in **(A, B)** and relative to the TREM-1 high population in **(D)**. Percentage of CD206+CD14+ cells of TREM-1 low or high CD14+ monocytes cultured with T-cells of a different donor in combination with anti-TNF (adalumimab, 10 µg/ml) or IgG isotype control (10 µg/ml). Representative plots **(E)**, quantified in (**F**, n=5 per condition) of 3 different independent experiments using different donors are shown. Whiskers indicate the 10-90 percentiles in **(A, B)** and de mean + SEM is shown in **(D, F)**. Significance was determined by a Mann Whitney **(A, B)**, paired-t-test **(D)** and ANOVA followed by Sidak’s multiple comparison test **(F)** with *P < 0.05, **P < 0.01, ***P < 0.001.

Frozen PBMCs from the CD patients were thawed and incubated with αCD3-APC(17-0038), αCD8-APCeF780(47-0087), CD14-PerCP-Cy5.5(45-0149), αCD19-PE(12-0199-80), αCD45-AF700 (56-9459)(all Thermo Fisher Scientific) and αCD4-BB515 (564419, BD Biosciences) at 4°C for 30 minutes. Before acquisition, samples were filtered through a 40 µm mesh cell strainer, and DAPI added to a final concentration of 0.1 µg/ml. CD14+ cells were sorted (BD FACSAria III), lysed (QIAshredder, Qiagen, Hilden, Germany) and stored at -80°C until RNA extraction. Freshly isolated PBMCs from healthy individuals were stained for αCD14-PE (Clone MφP9, BD Biosciences 345785) and αTREM-1-AF647(clone 193015, BD Biosciences Pharmingen 564472) and CD14 TREM-1 low and high monocytes were sorted on a SONY SH800S or BD FACS ARIA III as shown in **Figure 1C**. After culture to determine regulatory macrophage differentiation cells were stained with αCD14-PE-CY7 (Clone 61D3, eBiosciences) in combination with αCD206-PE-CF594 (Clone 19.2, BD Horizon). The mixed lymphocyte reactions (MLRs) were stained with αCD14-PE and αCD206-APC (Clone 19.2, BD Pharmingen) or αCD14-PE, αCD206-PE-CF594 and αTREM-1-AF647. For analysis of FcγR levels monocytes were stained with αCD14-PE-CY7 and αTREM-1-AF647 in combination with αFcyRI-PE (clone 10.1, Sony 2125040), αFcyRII-PE (clone 2E1, Immunotech 1935), αFcγRIII-PE (clone 3G8, BD Pharmingen 555407), or relevant isotype controls (IgG1, clone X40, BD Pharmingen 340013 and IgG2, Clone MOPC-173 Biolegend 400214). Cells were analyzed using a FACS Fortessa (BD) and FlowJo software (Treestar Inc., Ashland, OR). The mean fluorescence intensity (MFI) was determined by subtracting the background MFI of isotype stained cells.

### RNA Isolation

Lysed CD14+ sorted cells (obtained from CD patients initiating anti-TNF therapy) were processed for RNA extraction using the AllPrep DNA/RNA Mini kit (Qiagen, Hilden, Germany), according to the manufacturer’s instructions. The mRNA from the TREM-1 low and TREM-1 high CD14+ monocytes from the healthy individuals was isolated by the ISOLATE II RNA Mini Kit (Bioline, QC-Biotech, Alphen ad Rijn, The Netherlands) or the Agencourt RNAdvance Cellv2 kit (A47942, Beckman Coulter, Woerden, The Netherlands) according to manufacturer’s conditions.

### *Gene* Expression Analysis

#### Quantitative RT-PCR

Complementary DNA was synthesized from mRNA using Oligo-dT (ThermoFischer Scientific), random hexamer primers (Promega, Madison, USA), RiboLock RNase and RevertAid reverse transcriptase (both ThermoFischer Scientific). Quantitative RT-PCR (qPCR) was performed on a CFX96™ Real-Time System (Bio-Rad Laboratories, Hercules, CA, USA) using Sensifast SYBR green (Bioline) and primers for *TREM-1, FCGR2B, FCGR3A (*quantitect primers, Qiagen*), TREM-1_MB, TREM-1_SV, TREM-1_X2 and bACTIN* [see Verstockt et al. (10)]. mRNA levels were normalized against *bACTIN* and gene expression was calculated with the 2^-Δct^ method using LinRegPCR (Amsterdam UMC).

#### Microarray

For microarray analysis of the TREM-1 low and TREM-1 high CD14+ monocytes the RNA quality (integrity and concentration) was measured on an Agilent 2100 Bioanalyzer. All samples had a RNA integrity number (RIN) above 9,0 and concentration above 250 pg/µl. Microarray analysis was performed using a Clariom S Pico Human Assay (#902929, Thermo Fisher Scientific) using a Gene Titan MC system (Affymetrix) according to the standard protocols of the Dutch Genomics Service and Support Provider (MAD, University of Amsterdam, Netherlands). The data was normalized using robust multiarray analysis and analyzed by Transcriptome Analysis Console software (Thermo Fisher Scientific).

### Westernblot

TREM-1 high and low FACSorted cells were lysed in RIPA buffer (0.15M NaCl, 0.05M TRIS pH 7.5, 1% NP40, 0.5% DCA, 0.1% SDS, 0.1 mM EDTA), run on 15% SDS-PAGE gels under reducing conditions and transferred to an Immobilon-P PVDF Membrane (Millipore, Burlington, MA). The membrane was blocked by incubation in 5% non-fat milk (Nutricia, Wageningen, The Netherlands) in TBST (TBS + 0.1% Tween-20) for 2 hours at room temperature (RT) and subsequently incubated in LC3 (Cell Signaling, 4108S) or Cathepsin S (Abcam, ab18822) antibodies in 2% milk/TBST overnight at 4° C. After incubation membranes were washed 3 times with TBST, incubated with HRP conjugated secondary antibodies (1:2000, DAKO) in 2% milk/PBST for 2 hours at RT. Expression was detected by Lumilight Plus (Roche, Woerden, The Netherlands). Afterwards blots were stripped in stripping buffer (ThermoFischer Scientific) for 10 min at RT and incubated with β-actin (clone AB1978, Sigma, Deisenhofen, Germany) as a loading control. The Optical Density (OD) of the protein bands was determined using ImageJ software.

### Statistical Analysis

Data were analyzed using Kruskal-Wallis followed by Dunn’s *post hoc* test, analysis of variance (ANOVA) followed by Sidak’s *post hoc*, Mann Whitney test, paired t-test, unpaired t-tests or Wilcoxon signed rank test using Graphpad Prism 8.3.0 (Graphpad Software Inc., La Jolla, CA, USA. Values of p<0.05 were considered significant with *p<0.05, **p<0.01, ***p<0.001, and ****p<0.0001.

## Results

### TREM-1 Expression in CD14+ Monocytes Predicts Anti-TNF Response

Pretreatment *TREM-1* expression levels of CD14+ monocytes in peripheral blood of CD patients were predictive of outcome to anti-TNF mAb therapy, with low *TREM-1* expression being associated with response to anti-TNF (**Figure 1A**). When analyzing the expression of the different *TREM-1* isoforms we found that *TREM-1_MB*, the full TREM-1 receptor isoform expressed on the cell membrane, was significantly increased in CD14+ monocytes of anti-TNF non-responders compared to responders, while the other *TREM-1* isoforms,*TREM-1_SV* and *TREM-1_X2*, tended to be increased significantly (**Figure 1B**). The serum sTREM-1 levels of these CD patients [partly published in (9)] were not significantly different between anti-TNF non-responders compared to responders (**Supplementary Figure S1A**). The serum sTREM-1 levels tended to correlate with the expression levels of *TREM-1_SV*, the specific splice variant that encodes sTREM-1 (20) (**Supplementary Figure S1B**). The absence of a correlation with total *TREM-1* or *TREM-1_MB* expression levels (**Supplementary Figure S1C**), that were predictive of anti-TNF response, indicate that circulating sTREM-1 levels do not directly contribute to the found association with anti-TNF response.

### *TREM-1* Expression Associates With Regulatory Macrophage Differentiation *In Vitro*

Differentiation of CD14+ monocytes to regulatory CD206+ M2 type macrophages, mediated through Fcγ-Receptor signaling, is part of the mechanism of action of anti-TNF mAbs in IBD (11, 13, 14). As especially the full TREM-1 receptor isoform,* TREM-1_MB*, was increased in CD14+ monocytes of anti-TNF non-responders compared to responders we hypothesized this full receptor form might be involved in the suppression of differentiation towards CD206+ M2 macrophages, hence suppress the response to anti-TNF. The anti-TNF induced differentiation of CD14+ monocytes can be studied quite simple *in vitro* by using a mixed lymphocyte reaction (MLR), an allogenic immune reaction created by mixing peripheral blood mononuclear cells (PBMCs) of two different donors, as extensively reported earlier (11, 13, 14). To investigate the relation between TREM-1 levels and CD14+ monocyte differentiation, we FACSorted CD14+ monocytes with different TREM-1 cell surface levels (low vs high, see **Figure 1C**), and cultured them with T-cells of a different donor, creating a allogenic immune reaction. As expected, in these sorted populations the *TREM-1* expression levels were significantly decreased in the low versus high population, especially the full receptor form *TREM-1_MB* (**Figure 1D**). The CD14+ monocytes with low TREM-1 expression levels were most capable of polarizing towards CD206+ regulatory macrophages in the presence of anti-TNF mAbs (**Figures 1E, F**).

### TREM-1 Signaling Does Not Suppress Regulatory Macrophage Differentiation

**Figure 2:**
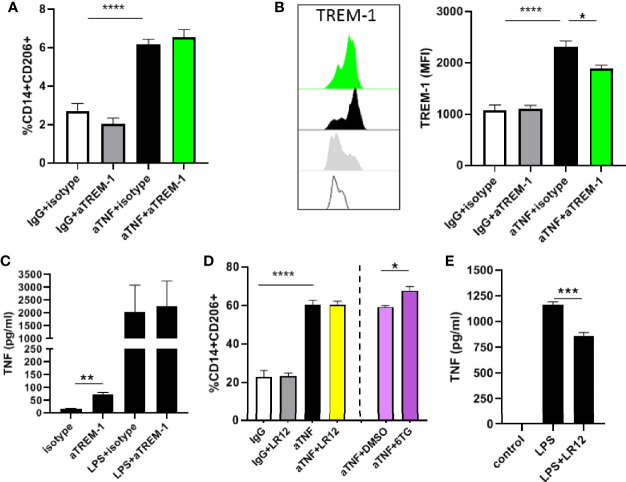
TREM-1 signaling does not affect anti-TNF induced differentiation of regulatory type macrophages. **(A)** Percentage of CD206+CD14+ cells in a mixed lymphocyte reaction with anti-TNF (adalumimab, 10 µg/ml), IgG isotype control (10 µg/ml) in combination with the agonistic TREM-1 antibody or relevant isotype control (both 10 µg/ml). **(B)** TREM-1 MFI of the CD14+CD206+ population in the MLR. **(D)** Percentage of CD206+CD14+ cells in a mixed lymphocyte reaction with anti-TNF, IgG isotype control in combination with the TREM inhibitor LR12 (50 µg/ml), and 6-TG (25 µM) or DMSO control. TNFα production by monocytes stimulated for 24 hours with the agonistic TREM-1 antibody (10 µg/ml) in **(C)** and with LPS (100 ng/ml) in combination with LR12 (50 µg/ml) in **(E)**. Representative (n=3-5 per condition) of 3 to 4 different independent experiments using different donors. Significance was determined by ANOVA followed by Sidak’s multiple comparison test in A/B/D and unpaired t-test in C/E with *P < 0.05, **P < 0.01, ***P < 0.001 and ****P < 0.0001.

As low TREM-1 levels are associated with increased induction of M2 differentiation by anti-TNF mAbs we wondered whether TREM-1 signaling would interfere with the induction of differentiation by anti-TNF mAbs. To test this we added a TREM-1 antibody with agonistic activity (16, 21) to anti-TNF mAbs in the MLR. There was no effect on the induction of CD206+ macrophages by anti-TNF by the agonist TREM-1 antibody (**Figure 2A**). The expression of TREM-1 was increased in the CD206+ macrophages that were induced by anti-TNF, and the detection of TREM-1 by flow cytometry was blocked by binding of the agonistic TREM-1 antibody (**Figure 2B**). To functionally show this antibody has TREM-1 agonistic activity monocytes were stimulated and released TNFα upon stimulation (**Figure 2C**), as reported (16, 21). The opposite experiment, in which we inhibited TREM-1 signaling, by the TREM-1 inhibitor LR12 (22, 23) also did not affect the anti-TNF-induced differentiation of CD14+ monocytes towards CD206+ macrophages in the MLR (**Figure 2D**). In this experiment 6-thioguanin (6TG) synergized with anti-TNF in the induction of CD14+CD206+ cells (**Figure 2A**), as we reported before (18), showing that the effect of anti-TNF could be enhanced in this experimental set-up. To show LR12 was effective in blocking TREM-1 signaling in the tested dose we again investigated TNFα production of monocytes and found LR12 was capable of decreasing TNFα production upon stimulation with LPS (**Figure 2E**) as reported (24). These experiments show that it is not TREM-1 signaling itself that is responsible for the different anti-TNF-induced differentiation of low versus high TREM-1 CD14+ monocytes, indicating something underlying discriminates these different populations of monocytes.

### TREM-1 Expression Associates With Autophagy

**Figure 3:**
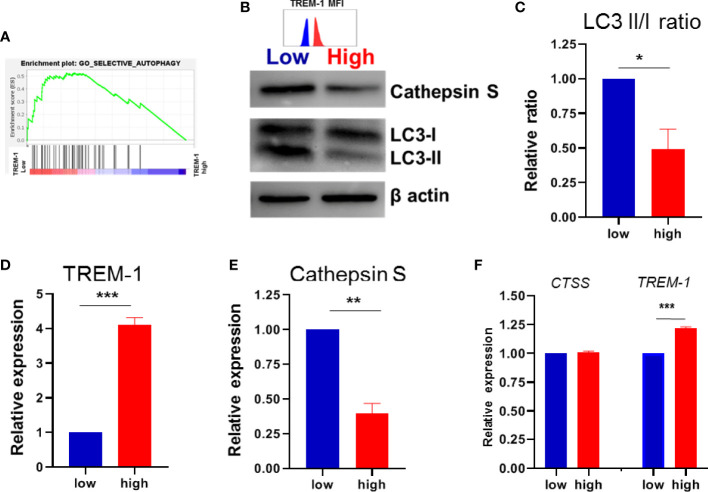
TREM-1 expression and autophagy. **(A)** GSEA plot of autophagy in low versus high TREM-1 CD14+ monocytes. **(B)** Immunoblot for LC3I and LC3II, Cathepsin S and b-actin as a loading control in paired low vs high TREM-1 CD14+ monocytes, representative of 4 different donors. TREM-1 MFI of the sorted populations is indicated above the blot The LC3II/I ratio, Cathepsin S and TREM-1 expression (MFI) were quantified in **(C–E)** and expressed relative to the low TREM-1 population. **(F)** *CTSS* gene and *TREM-1* expression from the array. Mean + SEM is shown in **(C–F)**. Significance was determined paired t-tests **(C–E)** and Wilcoxon signed rank tests in **(F)** with *P < 0.05, **P < 0.01, ***P < 0.0001.

We tried to elucidate which underlying pathway(s) is/are responsible for the found difference in anti-TNF-induced differentiation between low versus high TREM-1 CD14+ monocytes. Therefore we performed gene expression analysis on FACSorted TREM-1 low versus TREM-1 high CD14+monocytes (**Supplementary Figure S1**). Gene Set Enrichment Analysis (GSEA) revealed that 14 of the 15 autophagy pathways were enriched in the low TREM-1 population versus the TREM-1 high population (**Figure 3A**/**Supplementary Figure S2**). The relation between TREM-1 and autophagy in IBD has been described before (25), and as we found that autophagy levels are increased upon the induction of regulatory type macrophages by anti-TNF we decided to investigate this further by immunoblotting for LC3. Indeed we found that LC3II/LC3I ratios, indicative of autophagy, were significantly lower in TREM-1 high monocytes compared to their TREM-1 low counterparts (**Figures 3B–D**). Levels of Cathepsin S, an autophagy related protein which was identified as an inducer of regulatory type macrophages by anti-TNF, was significantly lower in the TREM-1 high versus low monocytes (**Figures 3B, E**), although the *CTSS* gene expression levels were not significantly different (**Figure 3F**). Lower levels of autophagy and Cathepsin S in CD14+ monocytes with high levels of TREM-1 could explain the reason for lower induction of regulatory type macrophages by anti-TNF.

### TREM-1 Expression Associates With Activation of the FcγR Pathway

**Figure 4:**
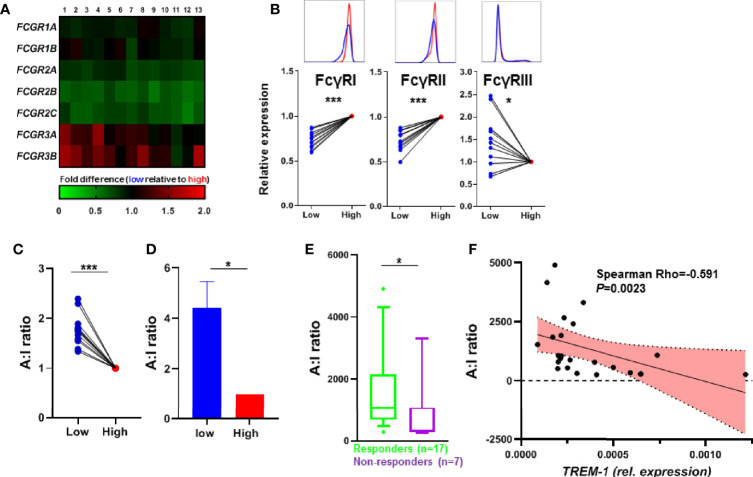
TREM-1 expression and FcγR signaling. **(A)** Heatmap of the expression of *FCGR* transcripts, paired expression of the TREM-1 low population relative to the TREM-1 high population of different donors (n=13). **(B)** The protein levels of FcγRI, FcγRII and FcγRIII were determined by flow cytometry. The MFI was corrected for isotype control on TREM-1 low and high CD14+ gated monocytes and expressed as the TREM-1 low population relative to TREM-1 high for each donor (n=12). **(C)** The FcγR A:I ratio determined by dividing the *FCGR3A* expression (activating) by the *FCGR2B* expression (inhibitory) of **(B)**, as suggested by Castro-Dopico *et al*. validated by the FcγR A:I ratio determined by *FCGR3A* and *FCGR2B* qPCR on the sorted samples from **Figure 1D** in **(D)** (n=4). The FcγR A:I ratio of the CD14+ monocytes from CD patients (n=24) before the start of anti-TNF therapy **(E)** and in correlation to the *TREM-1* expression levels **(G)**. Mean + SEM is shown in **(D)**, whiskers indicate the 10-90 percentiles in **(F)**. Significance was determined by Wilcoxon signed rank tests **(B, C)**, a paired t-test **(D)**, and Mann Whitney **(E)** with *P < 0.05, ***P < 0.001.

Besides the autophagy pathway also the FcγR pathway was enriched in the TREM-1 low population (**Supplementary Figure S3**), which became even more apparent when we compared paired samples (so low vs high TREM-1 samples of the same individual, **Figure 4A**), We confirmed the differential FcγR protein levels in TREM1- low vs TREM-1 high CD14+ monocytes by flow cytometry (**Figure 4B**). The activating FcγR gene transcript *FCGR3A*, essential for the induction of regulatory type macrophages by anti-TNF (13), was increased in low vs high TREM-1 expressing CD14+ monocytes (**Figure 4A**), while the inhibitory receptor *FCGR2B* was decreased in low vs high TREM-1 expressing CD14+ monocytes. Recently Castro-Dopico et al. (26) used the expression levels of *FCGR3A* and *FCGR2B*, to calculate a FcγR activating:inhibitory (A:I) ratio, and showed that this FcγR A:I ratio is altered in IBD. Calculating this FcγR A:I ratio showed it was significantly increased in TREM-1 low versus TREM-1 high CD14+ monocytes (**Figures 4C, D**). Indeed when investigating this FcγR A:I ratio in the CD14+ monocytes of the CD patients we found it was significantly higher in the patients that responded to anti-TNF (**Figure 4E**), and was negatively correlated with the *TREM-1* levels in the CD14+ monocytes of the CD patients (**Figure 4F**).

## Discussion

The whole blood *TREM-1* expression has been described as a predictive marker for anti-TNF mAb therapy responsiveness in patients with IBD (3, 9, 10) Interestingly, while both Gaujoux et al. (3) and Verstockt et al. (10) describe *TREM-1* expression consistently being upregulated in intestinal biopsies of anti-TNF non-responders compared to anti-TNF responders, the high *TREM-1* expression in whole blood was only found to associate with anti-TNF non-response in the study by Verstockt et al. (10), in contrast to the study by Gaujoux et al. (3) that showed exactly the opposite association. Although the difference in patient populations, difference in response criteria and the different primers used to determine *TREM-1* expression could explain the found differences, mainly the lack of understanding the underlying biological mechanism of the association of whole blood *TREM-1* expression specifically with anti-TNF response makes its use in clinical practice questionable. Here we set out to investigate the reason for the predictive value of *TREM-1* expression in whole blood for anti-TNF responsiveness in IBD. Therefore we first analyzed the sole expression of *TREM-1* in CD14+ monocytes, one of the main cell types in blood that expresses TREM-1 abundantly (16) and is important in mediating efficacy of anti-TNF therapy (11, 13, 14, 17). We found that low *TREM-1* levels in CD14+ monocytes isolated before anti-TNF treatment were associated with response to anti-TNF. Deciphering the reason for this association we investigated the functional consequence for CD14+ monocytes to express low or high levels of *TREM-1*. TREM-1 protein levels on the cell surface of CD14+ monocytes were found to correlate with their ability to differentiate into M2 macrophages upon anti-TNF mAb treatment, while TREM-1 signaling was not involved. Characterization of underlying pathways revealed that the autophagy pathway was increased in the CD14+ monocytes with low TREM-1 levels which we confirmed on the protein level. The association between TREM-1 signaling and autophagy in IBD has been described (25), and is associated with the induction of regulatory macrophages by anti-TNF (15). In addition, the activating FcγR pathway was increased in the CD14+ monocytes with low TREM-1 levels, which we have identified to be crucial for the response to anti-TNF (13). Moreover, not only were the FcγR activity levels in CD14+ monocytes of the CD patients negatively correlated with the *TREM-1* levels, also the high pretreatment FcγR activity levels were predictive of response to anti-TNF. As we identified both pathways earlier to be essential for the induction of M2 macrophages by anti-TNF mAb therapy, this might explain the found differences. It would indicate that CD14+ monocytes with high TREM-1 levels have a lower FcγR activation threshold signaling when an anti-TNF-TNF immune complex is encountered, important for the response (11, 13), while decreased autophagy levels would contribute to the suppression of the subsequent differentiation towards regulatory type macrophages. Another mechanism of action of anti-TNF therapy that has been proposed is the induction of T-cell apoptosis through various mechanisms (27). Transmembrane TNFα (mTNFα) expressed on macrophages can protect T-cells from undergoing apoptosis through interaction with the TNF-Receptor 2 (TNF-R2), and interference of this signal by anti-TNF induces T-cell apoptosis (17). Apoptosis resistant T-cells expressing both TNF-R2 and IL-23 Receptor were significantly more present the intestine of anti-TNF non-responders and could be activated by IL-23 produced by CD14+ macrophages (28). Although we have not specifically investigated these pathways we did not detect differences in *TNFA* or *IL23* expression in relation to *TREM-1* expression in the CD14+ monocytes (not shown). In conclusion, our results indicate that *TREM-1* expression levels in CD14+ monocytes associate with both decreased FcγR activity and autophagy levels resulting in decreased differentiation to M2 type regulatory macrophages upon anti-TNF mAb treatment, which may explain anti-TNF non-response in IBD patients with high expression levels of *TREM-1*.

## Data Availability Statement

The datasets presented in this study can be found in online repositories. The names of the repository/repositories and accession number(s) can be found below: https://www.ncbi.nlm.nih.gov/, GSE165522.

## Ethics Statement

The studies involving human participants were reviewed and approved by Institutional Review Board approved IBD Biobank of University Hospitals Leuven, Belgium (B322201213950/S53684) and The Medical Ethical Committee of the Amsterdam UMC, METC 2009_113). The patients/participants provided their written informed consent to participate in this study.

## Author Contributions

MP, BV, MW and PK performed experiments and analyzed and discussed data. MF and SV provided patient samples and discussed data. PK and MW supervised the study. MP and PK wrote the manuscript with input of all authors. All authors contributed to the article and approved the submitted version.

## Funding

Part of this research has been funded by the European Crohn’s and Colitis Organization Research Grant and by the Belgian IBD Research and Development group Research Grant (both to BV).

## Conflict of Interest

MF received research grants from Amgen, Biogen, Janssen, Pfizer, Takeda, consultancy fees from Abbvie, Boehringer-Ingelheim, Janssen, MSD, Pfizer, Sandoz, Takeda, and speakers fees from Abbvie, Amgen, Biogen, Boehringer-Ingelheim, Falk, Ferring, Janssen, Lamepro, MSD, Mylan, Pfizer, Takeda. SV received research grants from MSD, AbbVie, Takeda, Pfizer, Janssen, consultancy fees from AbbVie, MSD, Takeda, Ferring, Genentech/Roche, Shire, Pfizer Inc, Galapagos, Mundipharma, Hospira, Celgene, Second Genome, Progenity, Lilly, Arena, GSK, Amgen, Ferring, Gilead and Janssen, and speakers fees from AbbVie, MSD, Takeda, Ferring, Hospira, Pfizer, Janssen, and Tillots. BV reports financial support for research from Pfizer; lecture fees from Abbvie, Biogen, Chiesi, Falk, Ferring, Galapagos, Janssen, MSD, Pfizer, R-Biopharm, Takeda and Truvion; consultancy fees from Janssen, Guidepont and Sandoz; all outside of the submitted work. MW received research grants from GlaxoSmithKline, Boehringer Ingelheim and Stryker and speakers fees from Takeda and Janssen.

The remaining authors declare that the research was conducted in the absence of any commercial or financial relationships that could be construed as a potential conflict of interest.

## Supplementary Material

Supplementary Figure 1 Serum sTREM-1 levels. **(A)** The serum sTREM-1 levels of the CD patients, part of the already published cohort (9). **(B)** Correlation between sTREM-1- levels and CD14+ monocyte *TREM-1_SV* expression. **(C)** Correlation between sTREM-1 and CD14+ monocyte expression of *TREM-1 and the isoforms TREM-1_MB and TREM-1_X2.*

Supplementary Figure 2 Top 50 different regulated genes. Top 50 different regulated genes in TREM-1 high vs TREM-1 low CD14+ monocytes (based on ANOVA p<0.05 no multiple corrections).

Supplementary Figure 3 Differential gene expression profiles of the autophagy pathway. Gene expression profiles of TREM-1 high and TREM-1 low CD14+ monocytes were analyzed for enrichment of 15 different autophagy pathways.

Supplementary Figure 4 Differential gene expression profiles of the FcγR pathway. Gene expression profiles of TREM-1 high and TREM-1 low CD14+ monocytes were analyzed for enrichment of 6 different FcγR pathways.

## References

[1] Roda G Jharap B Neeraj N Colombel JF. Loss of response to anti-tnfs: Definition, epidemiology, and management. Clin Trans Gastroenterol (2016) 7(1):e135. 10.1038/ctg.2015.63 PMC4737871 26741065

[2] Naviglio S Giuffrida P Stocco G Lenti MV Ventura A Corazza GR. How to predict response to anti-tumour necrosis factor agents in inflammatory bowel disease. Expert Rev Gastroenterol Hepatol (2018) 12(8):797–810. 10.1016/j.arr.2018.11.003 29957083

[3] Gaujoux R Starosvetsky E Maimon N Vallania F Bar-Yoseph H Pressman S. Cell-centred meta-analysis reveals baseline predictors of anti-tnfalpha non-response in biopsy and blood of patients with ibd. Gut (2018). 10.1136/gutjnl-2017-315494 PMC6580771 29618496

[4] Czarnewski P Parigi SM Sorini C Diaz OE Das S Gagliani N. Conserved transcriptomic profile between mouse and human colitis allows unsupervised patient stratification. Nat Commun (2019) 10(1):2892. 10.1038/s41467-019-10769-x 31253778 PMC6598981

[5] West NR Hegazy AN Owens BMJ Bullers SJ Linggi B Buonocore S. Oncostatin m drives intestinal inflammation and predicts response to tumor necrosis factor-neutralizing therapy in patients with inflammatory bowel disease. Nat Med (2017) 23(5):579–89. 10.1038/nm.4307 PMC5420447 28368383

[6] Arijs I Li K Toedter G Quintens R Van Lommel L Van Steen K. Mucosal gene signatures to predict response to infliximab in patients with ulcerative colitis. Gut (2009) 58(12):1612–9. 10.1136/gut.2009.178665 19700435

[7] Arijs I Quintens R Van Lommel L Van Steen K De Hertogh G Lemaire K. Predictive value of epithelial gene expression profiles for response to infliximab in crohn’s disease. Inflamm Bowel Dis (2010) 16(12):2090–8. 10.1002/ibd.21301 20848504

[8] Verstockt B Verstockt S Creyns B Tops S Van Assche G Gils A. Mucosal il13ra2 expression predicts nonresponse to anti-tnf therapy in crohn’s disease. Aliment Pharmacol Ther (2019) 49(5):572–81. 10.1111/apt.15126 PMC6849553 30663072

[9] Verstockt B Verstockt S Blevi H Cleynen I de Bruyn M Van Assche G. Trem-1, the ideal predictive biomarker for endoscopic healing in anti-tnf-treated crohn’s disease patients? Gut (2018). 10.1136/gutjnl-2018-316845 30007919

[10] Verstockt B Verstockt S Dehairs J Ballet V Blevi H Wollants WJ. Low trem1 expression in whole blood predicts anti-tnf response in inflammatory bowel disease. EBioMedicine (2019) 40:733–42. 10.1016/j.ebiom.2019.01.027 PMC6413341 30685385

[11] Bloemendaal FM Koelink PJ van Schie KA Rispens T Peters CP Buskens CJ. Tnf-anti-tnf immune complexes inhibit il-12/il-23 secretion by inflammatory macrophages via an fc-dependent mechanism. J Crohns Colitis (2018). 10.1093/ecco-jcc/jjy075 29860435

[12] McRae BL Levin AD Wildenberg ME Koelink PJ Bousquet P Mikaelian I. Fc receptor-mediated effector function contributes to the therapeutic response of anti-tnf monoclonal antibodies in a mouse model of inflammatory bowel disease. J Crohns Colitis (2016) 10(1):69–76. 10.1093/ecco-jcc/jjv179 26429698

[13] Bloemendaal FM Levin AD Wildenberg ME Koelink PJ McRae BL Salfeld J. Anti-tumor necrosis factor with a glyco-engineered fc-region has increased efficacy in mice with colitis. Gastroenterology (2017). 10.1053/j.gastro.2017.07.021 28756234

[14] Vos AC Wildenberg ME Duijvestein M Verhaar AP van den Brink GR Hommes DW. Anti-tumor necrosis factor-alpha antibodies induce regulatory macrophages in an fc region-dependent manner. Gastroenterology (2011) 140(1):221–30. 10.1053/j.gastro.2010.10.008 20955706

[15] Levin AD Koelink PJ Bloemendaal FM Vos AC D’Haens GR van den Brink GR. Autophagy contributes to the induction of anti-tnf induced macrophages. J Crohns Colitis (2016) 10(3):323–9. 10.1093/ecco-jcc/jjv174 PMC4957464 26417049

[16] Bouchon A Dietrich J Colonna M. Cutting edge: Inflammatory responses can be triggered by trem-1, a novel receptor expressed on neutrophils and monocytes. J Immunol (Baltimore Md 1950) (2000) 164:4991–5. 10.4049/jimmunol.164.10.4991 10799849

[17] Atreya R Zimmer M Bartsch B Waldner MJ Atreya I Neumann H. Antibodies against tumor necrosis factor (tnf) induce t-cell apoptosis in patients with inflammatory bowel diseases via tnf receptor 2 and intestinal cd14(+) macrophages. Gastroenterology (2011) 141(6):2026–38. 10.1053/j.gastro.2011.08.032 21875498

[18] Wildenberg ME Levin AD Ceroni A Guo Z Koelink PJ Hakvoort TBM. Benzimidazoles promote anti-tnf mediated induction of regulatory macrophages and enhance therapeutic efficacy in a murine model. J Crohns Colitis (2017). 10.1093/ecco-jcc/jjx104 PMC5881671 28961920

[19] Schnitzler F Fidder H Ferrante M Noman M Arijs I Van Assche G. Mucosal healing predicts long-term outcome of maintenance therapy with infliximab in crohn’s disease. Inflamm Bowel Dis (2009) 15(9):1295–301. 10.1002/ibd.20927 19340881

[20] Baruah S Keck K Vrenios M Pope MR Pearl M Doerschug K. Identification of a novel splice variant isoform of trem-1 in human neutrophil granules. J Immunol (Baltimore Md 1950) (2015) 195(12):5725–31. 10.4049/jimmunol.1402713 PMC4670805 26561551

[21] Bouchon A Facchetti F Weigand MA Colonna M. Trem-1 amplifies inflammation and is a crucial mediator of septic shock. Nature (2001) 410:1103–7. 10.1038/35074114 11323674

[22] Derive M Boufenzer A Gibot S. Attenuation of responses to endotoxin by the triggering receptor expressed on myeloid cells-1 inhibitor lr12 in nonhuman primate. Anesthesiology (2014) 120:935–42. 10.1097/ALN.0000000000000078 24270127

[23] Derive M Bouazza Y Sennoun N Marchionni S Quigley L Washington V. Soluble trem-like transcript-1 regulates leukocyte activation and controls microbial sepsis. J Immunol (Baltimore Md 1950) (2012) 188(11):5585–92. 10.4049/jimmunol.1102674 PMC6382278 22551551

[24] Dubar M Carrasco K Gibot S Bisson C. Effects of porphyromonas gingivalis lps and lr12 peptide on trem-1 expression by monocytes. J Clin Periodontol (2018) 45:799–805. 10.1111/jcpe.12925 29779263

[25] Kökten T Gibot S Lepage P D’Alessio S Hablot J Ndiaye NC. Trem-1 inhibition restores impaired autophagy activity and reduces colitis in mice. J Crohns Colitis (2018) 12(2):230–44. 10.1093/ecco-jcc/jjx129 28961797

[26] Castro-Dopico T Dennison TW Ferdinand JR Mathews RJ Fleming A Clift D. Anti-commensal igg drives intestinal inflammation and type 17 immunity in ulcerative colitis. Immunity (2019) 50(4):1099–114.e10. 30876876 10.1016/j.immuni.2019.02.006 PMC6477154

[27] Levin AD Wildenberg ME van den Brink GR. Mechanism of action of anti-tnf therapy in inflammatory bowel disease. J Crohns Colitis (2016) 10:989–97. 10.1093/ecco-jcc/jjw053 26896086

[28] Schmitt H Billmeier U Dieterich W Rath T Sonnewald S Reid S. Expansion of il-23 receptor bearing tnfr2+ t cells is associated with molecular resistance to anti-tnf therapy in crohn’s disease. Gut (2018). 10.1136/gutjnl-2017-315671 PMC6580782 29848778

