# Supplementary figures and images for: Monocyte TREM-1 Levels Associate With Anti-TNF Responsiveness in IBD Through Autophagy and Fcγ-Receptor Signaling Pathways
Source: Front Immunol. 2021 Mar 15;12:627535. doi: 10.3389/fimmu.2021.627535 (PMC8005579; doi:10.3389/fimmu.2021.627535)

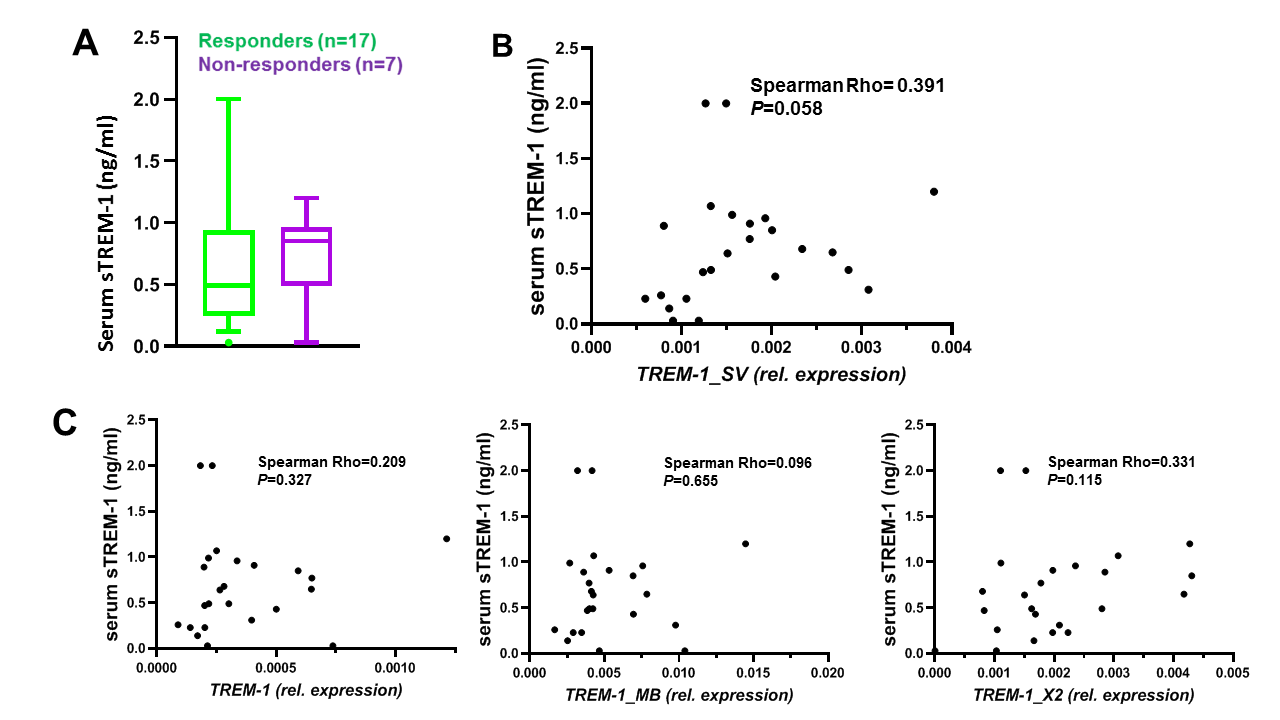

Supplement: Supplementary Figure 1 — Serum sTREM-1 levels. (A) The serum sTREM-1 levels of the CD patients, part of the already published cohort (9). (B) Correlation between sTREM-1- levels and CD14+ monocyte TREM-1_SV expression. (C) Correlation between sTREM-1 and CD14+ monocyte expression of TREM-1 and the isoforms TREM-1_MB and TREM-1_X2. [file Image_1.tif]

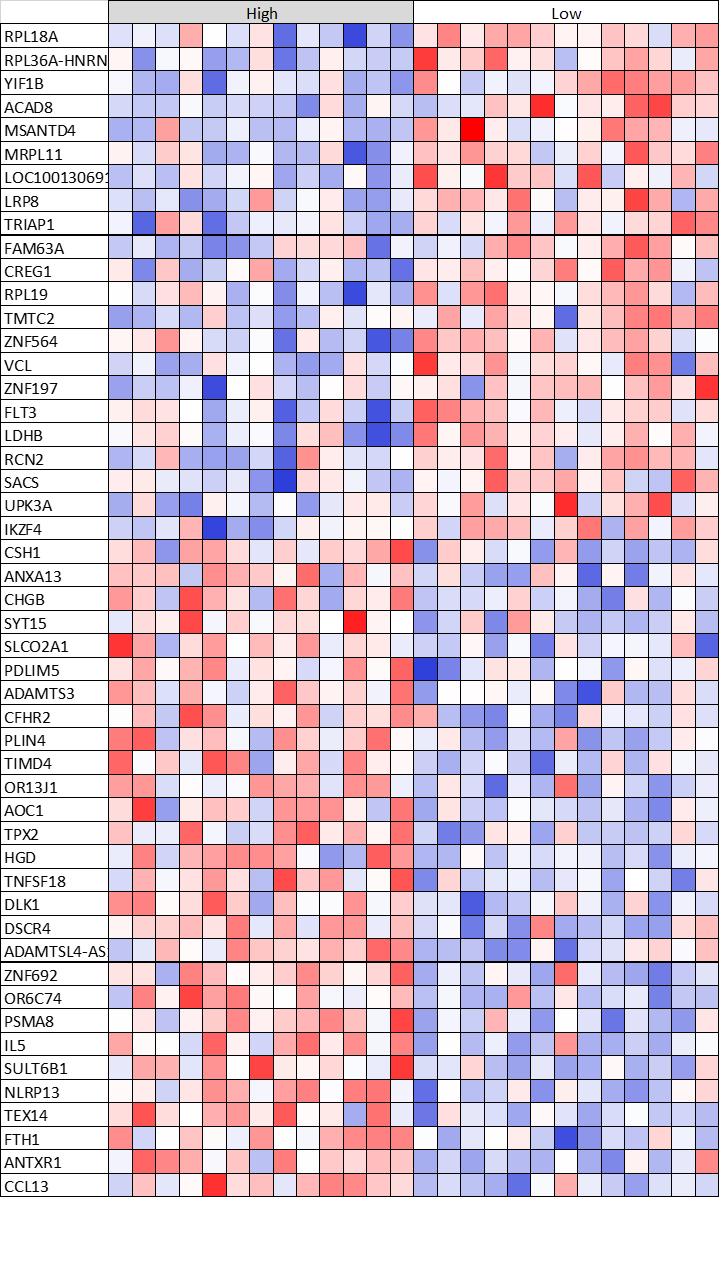

Supplement: Supplementary Figure 2 — Top 50 different regulated genes. Top 50 different regulated genes in TREM-1 high vs TREM-1 low CD14+ monocytes (based on ANOVA p<0.05 no multiple corrections). [file Image_2.tif]

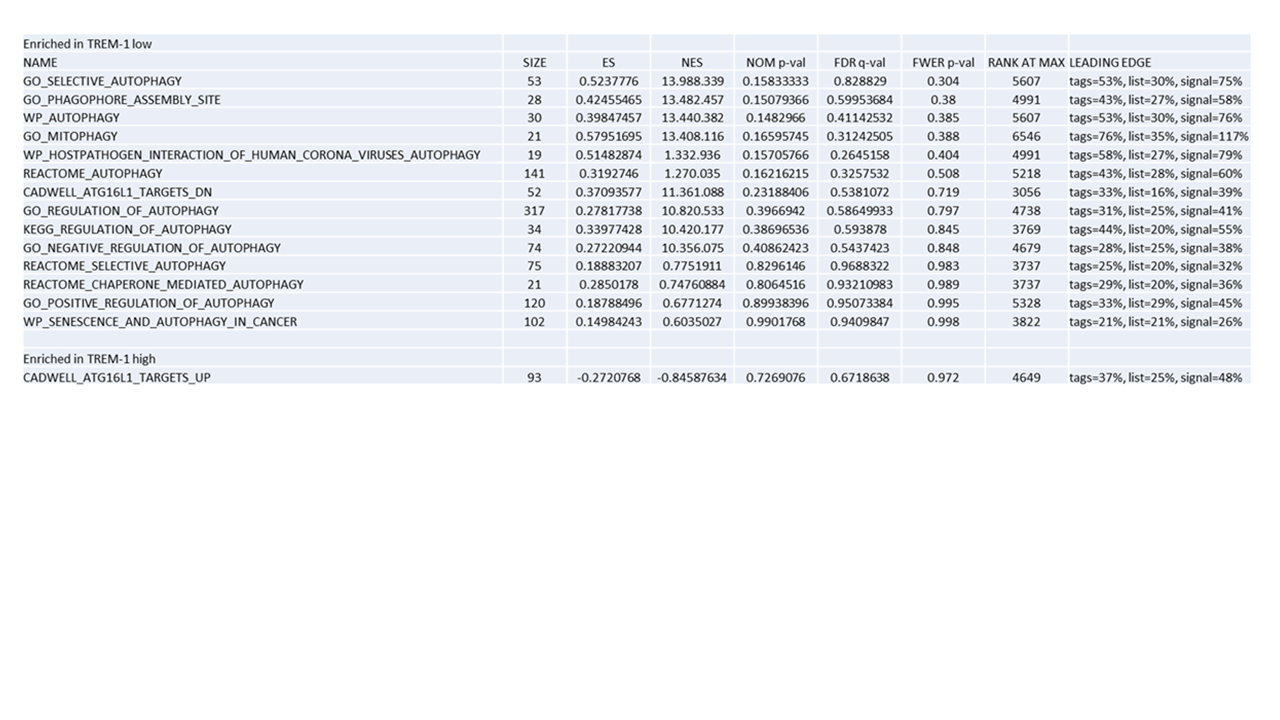

Supplement: Supplementary Figure 3 — Differential gene expression profiles of the autophagy pathway. Gene expression profiles of TREM-1 high and TREM-1 low CD14+ monocytes were analyzed for enrichment of 15 different autophagy pathways. [file Image_3.tif]

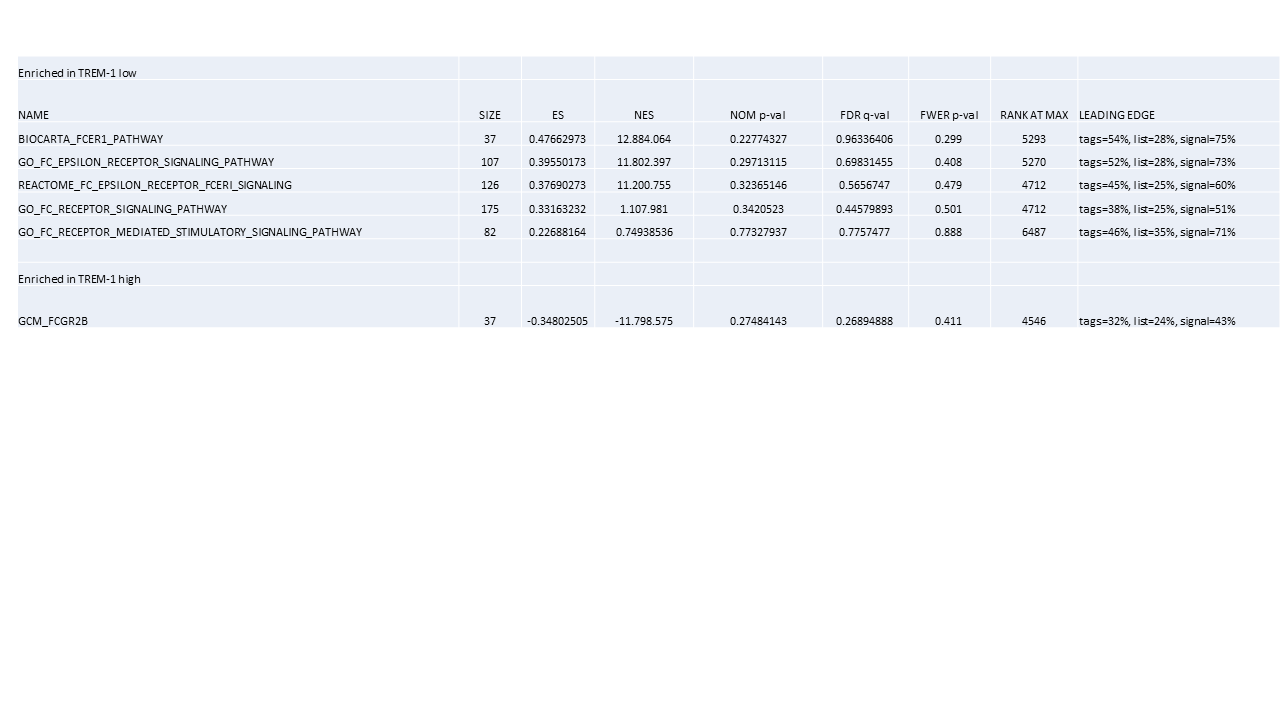

Supplement: Supplementary Figure 4 — Differential gene expression profiles of the FcγR pathway. Gene expression profiles of TREM-1 high and TREM-1 low CD14+ monocytes were analyzed for enrichment of 6 different FcγR pathways. [file Image_4.tif]
